# Depth-Differentiation and Seasonality of Planktonic Microbial Assemblages in the Monterey Bay Upwelling System

**DOI:** 10.3389/fmicb.2020.01075

**Published:** 2020-05-25

**Authors:** Linta Reji, Bradley B. Tolar, Francisco P. Chavez, Christopher A. Francis

**Affiliations:** ^1^Department of Earth System Science, Stanford University, Stanford, CA, United States; ^2^Biological Oceanography Group, Monterey Bay Aquarium Research Institute, Moss Landing, CA, United States

**Keywords:** Monterey Bay, coastal ocean, upwelling, microbial communities, SAR11, Cyanobacteria

## Abstract

Coastal upwelling regions are hotspots of biological productivity, supporting diverse communities of microbial life and metabolisms. Monterey Bay (MB), a coastal ocean embayment in central California, experiences seasonal upwelling of cold, nutrient-rich waters that sustain episodes of high phytoplankton production in surface waters. While productivity in surface waters is intimately linked to metabolisms of diverse communities of Archaea and Bacteria, a comprehensive understanding of the microbial community in MB is missing thus far, particularly in relation to the distinct hydrographic seasons characteristic of the MB system. Here we present the results of a 2-year microbial time-series survey in MB, investigating community composition and structure across spatiotemporal gradients. In deciphering these patterns, we used unique sequence variants (SVs) of the 16S rRNA gene (V4–V5 region), complemented with metagenomes and metatranscriptomes representing multiple depth profiles. We found clear depth-differentiation and recurring seasonal abundance patterns within planktonic communities, particularly when analyzed at finer taxonomic levels. Compositional changes were more pronounced in the upper 0–40 m of the water column, whereas deeper depths were characterized by temporally stable populations. In accordance with the dynamic nutrient profiles, the system appears to change from a Bacteroidetes- and Rhodobacterales-dominated upwelling period to an oceanic season dominated by oligotrophic groups such as SAR11 and picocyanobacteria. The cascade of environmental changes brought about by upwelling and relaxation events thus impacts microbial community structure in the bay, with important implications for the temporal variability of nutrient and energy fluxes within the MB ecosystem. Our observations emphasize the need for continued monitoring of planktonic microbial communities in order to predict and manage the behavior of this sensitive marine sanctuary ecosystem, over projected intensification of upwelling in the region.

## Introduction

Upwelling-influenced coastal regions that experience seasonal changes in productivity have been studied extensively, particularly in relation to phytoplankton community dynamics (e.g., Kudela et al., 2008; Lamont et al., 2014; also reviewed in Chavez and Messié, 2009; Capone and Hutchins, 2013). While phytoplankton comprise the principal source of dissolved organic matter (DOM) in coastal systems (Biddanda and Benner, 1997; Thornton, 2014), much of the carbon and energy fluxes through cascading trophic interactions are mediated by metabolically and phylogenetically diverse clades of Archaea and Bacteria (Jiao et al., 2010). As indispensable regulators of key biogeochemical processes, characterizing the dynamics of planktonic microbial populations in the coastal ocean is paramount to understanding ecosystem responses to environmental change (Doney et al., 2012).

Seasonality in microbial community structure has been examined in several marine environments, including in upwelling-influenced coastal systems (e.g., Fuhrman et al., 2006; Giovannoni and Vergin, 2012; Chow et al., 2013; Lindh et al., 2014; Alonso-Saez et al., 2015; Cram et al., 2015; Hernando-Morales et al., 2018; Lambert et al., 2019). Long term time-series sampling of microbial communities in several ocean regions have revealed recurring patterns in community composition, often with substantial depth-based variations in the temporal dynamics (e.g., Steele et al., 2011; Chow et al., 2013; Cram et al., 2015; Hernando-Morales et al., 2018). In dynamic coastal regions, such periodic and deterministic changes in community structure has been linked to seasonal phytoplankton blooms (e.g., Needham and Fuhrman, 2016; Teeling et al., 2016; Camarena-Gómez et al., 2018; also reviewed in Bunse and Pinhassi, 2017).

Monterey Bay (herein MB), a deep, non-estuarine embayment in central California, experiences wind-driven seasonal upwelling in spring/summer, supporting massive phytoplankton blooms. The highly dynamic hydrography is reflected in the system fluctuating from high primary production (PP) during spring/summer to relatively low PP levels during non-upwelling periods (Chavez, 1996). Based on temporally varying hydrographic conditions, three oceanographic seasons have been defined for the MB system (Skogsberg, 1936; Bolin and Abbott, 1960): (i) a spring/summer ‘upwelling season’ from about March through July, (ii) a summer/fall ‘oceanic season’ during August/September through November and (iii) the winter Davidson Current period from December through February (also reviewed in Pennington and Chavez, 2000). In spring and summer, newly upwelled water supports massive phytoplankton blooms in the bay. During the post-upwelling relaxation period (the oceanic season), California Current water is advected onshore to replace the sinking upwelled water, causing temperature maxima in the surface resulting in thermal stratification. Occasional dinoflagellate-dominated blooms are observed during this period (Garrison, 1979; Schrader, 1981), which is often supported by fall upwelling events. In winter, the pole-ward flowing Davidson Current surfaces, leading to relatively warmer, less stratified waters.

In accordance with the seasonal changes in hydrology, predictable seasonal succession of phytoplankton populations has been a well-recognized feature of the bay ecology (e.g., Schrader, 1981). Although phytoplankton blooms are known to affect seasonal shifts in bacterial community composition (reviewed in Bunse and Pinhassi, 2017), very little is known about the bacterial and archaeal components of the MB system.

Prior studies examining planktonic microbial communities in MB have either focused on specific functional groups such as nitrifiers or picocyanobacteria (e.g., O’Mullan and Ward, 2005; Mincer et al., 2007; Paerl et al., 2012; Smith et al., 2014), or used targeted approaches with limited spatiotemporal resolution to characterize abundant marine bacteria (Suzuki et al., 2004; Rich et al., 2011; Nowinski et al., 2019). Such targeted surveys using bacterial artificial chromosome (BAC) libraries or oligonucleotide microarrays have led to exciting and important discoveries such as the discovery of proteorhodopsin phototrophy in surface waters and the responses of abundant bacterial clades to upwelling in the bay (Béjà et al., 2001; Rich et al., 2011). However, a holistic picture of planktonic microbial community dynamics in the MB system has been missing, particularly in the context of the bay seasonality. Pronounced seasonal patterns are generally observed for planktonic microorganisms in marine ecosystems, often related to variability in environmental conditions and nutrient availability (reviewed in Bunse and Pinhassi, 2017). Given the dynamic hydrology characteristic of seasonal transitions in MB, the lack of a seasonal succession signal in previous community surveys is indeed surprising.

Another major consideration concerning the bay ecology is the projected intensification of upwelling and nutrient fluxes in the California Current system, causing a cascade of ecological changes that may enhance shallow water hypoxia in the region (Xiu et al., 2018). In this context, a question with critical implications for biogeochemistry of the bay is how these projected changes would manifest on microbial ecology. Extrapolating and predicting community dynamics requires an understanding of the distribution of planktonic microbial assemblages along the temperature-salinity-nutrient gradients in MB, as well as their responses to the dynamic hydrographic conditions.

In this work, we combined high-throughput 16S rRNA amplicon sequencing, metagenomic, and metatranscriptomic approaches with extensive spatiotemporal sampling resolution to characterize microbial assemblages across physicochemical gradients in the MB system. Our results suggest strong depth partitioning and sequential dynamics among bacterial and archaeal clades, intimately linked to the seasonal transitions at MB.

## Materials and Methods

### Sample Collection and Sequencing

Water samples for nucleic acid extraction were collected from two stations in Monterey Bay (Supplementary Figure S1): M1 (36.747°N, −122.022°W; ∼26 km from shore) and M2 (36.697°N, −122.378 °W; ∼72 km from shore). These stations were sampled monthly between 2014 and 2016. In 2014, sampling was carried out at 5, 20, 30, 40, 80, 100, and 200 m depths. In 2015 and 2016, the 500 m depth was also sampled. Due to logistical reasons, sampling was not carried out in winter 2014.

Note that the sampling time coincided with a particularly warm period in the northeastern Pacific (Bond et al., 2015; Di Lorenzo and Mantua, 2016), which altered the phytoplankton ecology of the bay (Ryan et al., 2017). Details of sample collection, environmental metadata collection, and nucleic acid extraction are provided in Reji et al. (2019b) and Tolar et al. (2020).

Amplicon, metagenome, and metatranscriptome sequencing were carried out as part of a Community Science Program (CSP) project with the DOE Joint Genome Institute (JGI). Universal (Bacteria and Archaea) primers 515F-Y (5′-GTGCCAGCMGCCGCGGTAA) and 926R (5′-CCGYCAATTYMTTTRAGTTT) were used for amplifying the V4-V5 region of the 16S rRNA gene (Parada et al., 2016). Details of amplicon, metagenome, and metatranscriptome sequencing are provided in prior publications (Reji et al., 2019a, b). In addition to the 10 paired metagenome and metatranscriptome samples analyzed in Reji et al. (2019a), we generated another 20 metagenomes from M1 spanning multiple time points in 2015.

### Amplicon Data Processing and Analysis

To infer exact sequence variants (SVs), demultiplexed, quality-filtered reads were processed through the DADA2 pipeline (DADA2 package v1.10.1; ref. Callahan et al., 2016) in R (v3.5.1; R Core Team, 2018). Since samples were distributed across 4 individual plates, it was necessary to remove the barcoded primer sequences from the reads prior to applying the core DADA2 pipeline. We used cutadapt (v1.18; Martin, 2011) to remove primers before filtering reads based on read quality. R1 and R2 reads were filtered at 250 and 200 bp, respectively, using the filterAndTrim command in DADA2 [other parameter arguments were: maxN = 0, maxEE = c(2,2), truncQ = 2, rm.phix = TRUE]. Error models were generated using 1.5 million reads each for the filtered R1 and R2 read sets, and these models were then used for sequence inference using the main DADA2 algorithm. Sequence tables from each DADA2 run (i.e., each plate) were combined, and chimeric sequences were removed using removeBimeraDenovo command. The assignTaxonomy command was used for taxonomic assignment (tryRC = TRUE, minBoot = 80), using the silva_nr_v132 training set as the reference database.

Further analyses of the SVs were performed using the phyloseq package (v1.26.1; McMurdie and Holmes, 2013) in R. The SV count table and taxonomy table from the DADA2 pipeline above were combined with physicochemical data into a single phyloseq object. SVs classified as originating from eukaryotes, chloroplast, and mitochondria were filtered out. Only 251 reads were retained from the October 2014 sample from 5 m at M1; therefore, this sample was removed from the dataset. Total read count for the remaining samples ranged from 37904 to 98299. The final phyloseq object contained 8842 SVs observed across 292 samples.

### Statistical Analyses

All statistical analyses were performed on the R software analysis platform (v3.5.2), primarily using the vegan package (Oksanen et al., 2019). Phyloseq functions were used to compute various alpha and beta diversity measures. Data were rarefied using “rarefy_even_depth” function in phyloseq (this removed 284 SVs; resampled with replacement). Chao1 and Simpson alpha diversity indices were computed using the function “estimate_richness.” Pairwise comparisons of alpha diversity between hydrographic seasons were performed using the Wilcoxon rank-sum test with Bonferroni correction. Depth groups were identified using hierarchical cluster analysis, using the function hclust with Bray–Curtis dissimilarities calculated using vegdist (package vegan, v2.5-4). Results were visualized using the heatmap.2 function in the gplots library (v3.0.3). Beta diversity ordinations were performed using the “ordinate” function in phyloseq. Canonical Analysis of Principal Coordinates (CAP; ref. Anderson and Willis, 2003) was used to relate environmental variables to community structure. When two variables were highly correlated (*R*^2^ > 0.9; Supplementary Figure S3), one of them was dropped from the analysis to reduce redundancy.

Analysis of similarity (ANOSIM, ref. Clarke, 1993; implemented in vegan v2.5-4) and PERMANOVA (Anderson, 2001, implemented using the vegan function adonis) were used to characterize the degree of similarity between community structure across seasons for different depth groups (see section “Results” for details). Homogeneity of dispersion among sample groups (i.e., seasons; ref. Anderson, 2006) was examined using the vegan function betadisper. The BIOENV procedure (Clarke and Ainsworth, 1993) was used to identify the set of environmental variables correlating maximally with the Bray–Curtis dissimilarity matrix computed across seasons for each depth layer (implemented in vegan using the ‘bioenv’ function).

### Metagenomic and Metatranscriptomic Analysis

Details on metagenome and metatranscriptome read processing and analysis are presented in Reji et al. (2019a). Taxonomic comparisons were made using the phylogenetic distribution tool in IMG. Functional analyses were carried out based on KEGG Orthology (KO) annotations. Normalized KO abundances (KO abundances for each gene/pathway normalized to total KO annotations in each sample) were used to compare abundance and expression of key genes for phototrophy, nitrogen and sulfur cycle transformations, and transport pathways. Individual genes included in each pathway analyzed here are summarized in Supplementary Table S1.

### Data Availability

Amplicon sequence data have been deposited in the NCBI Sequence Read Archive (SRA) database under BioProject PRJNA488312 (SRA accession: SRP159037). All metagenomic and metatranscriptomic assemblies presented in this study are available in IMG, and can be found under the GOLD Study ID Gs0099541.

## Results

### Community Structure Varies by Depth and Hydrographic Season

During the roughly 2-year study period, physicochemical variables changed substantially across seasons at both stations. Temperature, salinity, and nitrate data were indicative of upwelling during spring and the subsequent relaxation period in fall (Supplementary Figure S4). As is typical of the MB system, the effect of upwelling on physicochemical parameters was most pronounced at depths above 100 m; temperature and nitrate levels showed minimal temporal variation at 200 and 500 m depths (Supplementary Figure S4).

**FIGURE 1:**
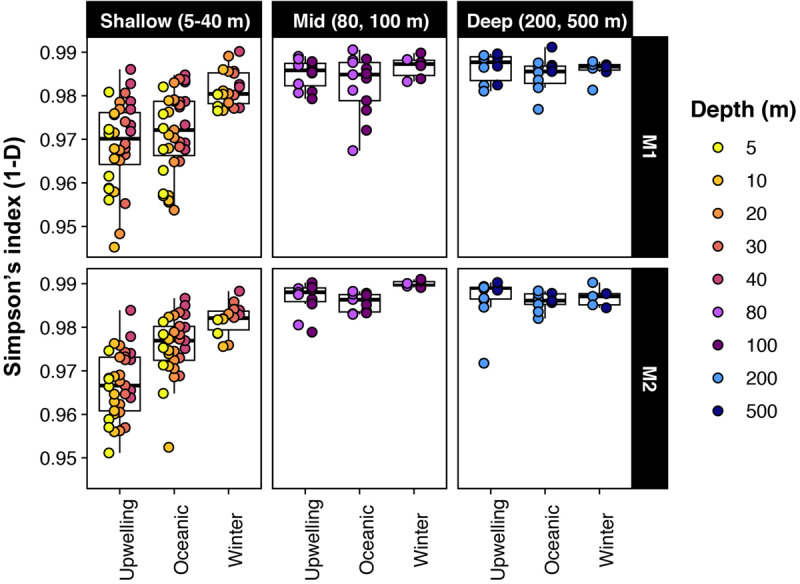
Alpha-diversity changed significantly across seasons in shallow depths; but was less variable in deeper waters. Note that Simpson’s index is presented as 1-D here (i.e., richness and evenness increases from 0 to 1).

Estimates of alpha diversity at the level of individual SVs were lowest at the shallow photic depths (5–40 m) and peaked at 80 m and below (Figure 1). Strong seasonal variation in richness and evenness was apparent in the shallow depths at both stations, whereas temporal changes were not as prominent below 40 m (Figure 1). Within the photic depths at M1, community alpha diversity in winter was significantly different from that in upwelling and oceanic seasons (estimated by comparing Simpson’s diversity index; pairwise Wilcoxon rank sum test with Bonferroni correction; *p*-value << 0.001); however, the diversity index did not change significantly between upwelling and oceanic seasons (*p*-value = 0.84). At comparable depths at M2, differences in Simpson’s index were significant between all three seasons, although relatively smaller in magnitude between oceanic and winter seasons (*p*-values < 1e−5; *p*-value for oceanic-winter comparison = 0.0056).

**FIGURE 2:**
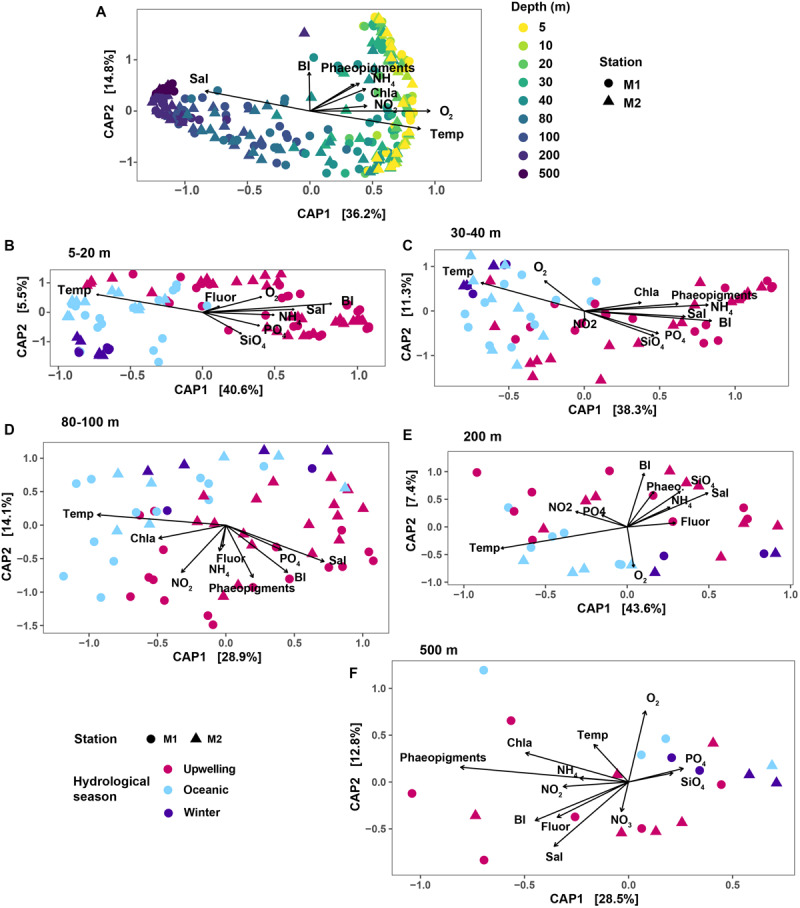
Results of CAP ordination analysis, relating SV abundances to measured environmental variables. In each plot panel, shapes correspond to the two stations. **(A)** CAP results on the overall dataset; colors indicate the various depths sampled. Remaining panels show results of CAP ordination on depth-subsetted data: **(B)** 5–20 m; **(C)** 30 and 40 m depths; **(D)** 80 and 100 m; **(E)** 200 m and **(F)** 500 m. In **(B–F)**, color mapping corresponds to the three hydrographic seasons in MB. The Bakun upwelling index was used as a proxy for bay seasonality. Variable abbreviations: BI, Bakun upwelling index; Chla, chlorophyll *a*; Fluor, fluorescence; Sal, salinity; Temp, temperature; Phaeo, phaeopigments.

Changes in microbial community structure correlated the most with water column depth (Figure 2A), as has been described previously for a subset of the time-series samples (Reji et al., 2019b). In the constrained analysis of principal components (CAP) performed on the rarefied SV dataset, CAP1 explained 36.2% of the variation, which primarily captured the strong depth gradient (Figure 2A). Temporal variability was more evident when the dataset was partitioned by depth. Within each depth layer (depth grouping was determined via hierarchical clustering as presented in Supplementary Figure S2), samples separated along a seasonal gradient with most distinct separation between upwelling and non-upwelling periods (Figures 2B–F). At shallow depths <30 m, temporal variability was more important in structuring the community than depth (Figure 2B; CAP1 captured seasonal variability in temperature and salinity, explaining 40.6% of the variability). While the influence of temperature on community structure appeared to be weaker at 500 m compared to the other depth groups, the primary axis of variation captured variability in phaeopigment levels (Figure 2F).

These results were further confirmed by ANOSIM performed on depth-subsetted SV abundance data (Table 1). At the shallow depths (5–40 m), ANOSIM results suggested significant differences in community structure across seasons (*R* = 0.562 for 5–20 m; and 0.593 at 30 and 40 m depths; *p* < 0.001 for both depth groups). Strength of the ANOSIM correlation decreased with depth (Table 1), further corroborating the ordination results that seasonal changes in hydrology differentially affect the shallow depths, with the strongest effects manifesting in the top 40 m of the water column. PERMANOVA results suggested similar depth-partitioned trends in community similarity across seasons (Table 1). Both the ANOSIM and PERMANOVA results for the shallow depth groups may be affected by dispersion effects, since the homogeneity of dispersions test rejected the null that group dispersions are the same for these two depth groups (Table 1).

**TABLE 1:** Results of statistical tests on seasonal changes in community structure.

	**Homogeneity of group**				**PERMANOVA**			
**Depth group**	**dispersions (betadisper)**		**ANOSIM**		**(adonis)**		**BIOENV**	
	***F* statistic**	***p*-value**	**Global R**	***p*-value**	***R*^2^**	***p*-value**	**Variables in best model**	**Spearman’s correlation**
5–30 m	7.29	0.001**	0.562	0.001	0.38	0.0009***	Bakun Index, Temperature	0.637
30–40 m	4.06	0.022*	0.593	0.001	0.38	0.0009***	Bakun Index, Temperature, Chlorophyll A, Ammonium	0.665
80–100 m	0.351	0.705	0.379	0.001	0.215	0.0009***	Temperature, Salinity, Nitrite	0.543
200 m	0.481	0.623	0.301	0.001	0.227	0.0009***	Temperature, Nitrite	0.563
500 m	1.347	0.286	0.227	0.007	0.236	0.0039**	Phaeopigments	0.574

Presented values include Analysis of similarities (ANOSIM), PERMANOVA (adonis), and BIOENV results. Asterisks indicate statistically significant results. *p ≤ 0.1, **p ≤ 0.01, and ***p ≤ 0.001.

A BIOENV rank-correlation analysis performed to identify variables (or combinations of variables) that best explain community dissimilarity at the SV-level identified temperature as an important explanatory variable across all depths except 500 m (Table 1). The seasonal upwelling signal was particularly evident at the top 40 m (Table 1). At 500 m, the best model contained the single predictor phaeopigment levels (*R*^2^ = 0.574) (Table 1).

### Taxa Contributing to Spatiotemporal Changes in Community Structure

Nearly half of all SVs in the dataset were classified as Proteobacteria at the phylum level (52% of the SVs). Comparing relative abundances of phyla, Proteobacteria comprised up to 60% of the community at both stations, with no discernible depth preferences at the phylum level (Supplementary Figure S5). All other major phyla, however, showed differential distribution patterns with depth (Supplementary Figure S5). Shallow waters (5–40 m) were dominated by Bacteroidetes, Verrucomicrobia and Euryarchaeota, while Thaumarchaeota, *Candidatus* Marinimicrobia (previously known as Marine Group A or SAR406), Planctomycetes and Nitrospinae became more abundant at 40 m or below (Supplementary Figure S5). Notable abundances of Thaumarchaeota and *Ca*. Marinimicrobia in shallow depths were observed only during the oceanic and winter seasons, primarily in 2015.

Temporal patterns in the relative abundances of many phyla, including Cyanobacteria, Bacteroidetes and Euryarchaeota, varied between the two stations. A temporal lag was apparent in the abundance of Cyanobacteria at 20–40 m depths at M2 compared to M1, particularly in 2014 (Supplementary Figure S5). Cyanobacteria also accounted for a slightly larger fraction of the microbial community at M2 in 2015, compared to similar time-points at M1 (Supplementary Figure S5). Planctomycetes were predominantly found below 40 m at both stations; however, during several of the non-upwelling time points at M1, they accounted for substantial fractions of the shallow depth communities (2–3% of the total; Supplementary Figure S5).

**FIGURE 3:**
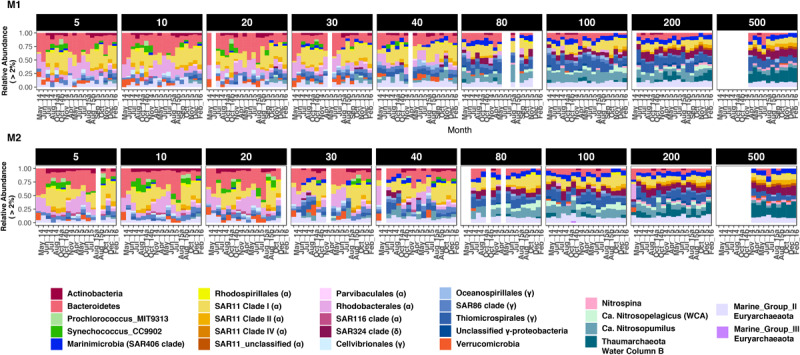
Bacterial and archaeal groups exhibited differential distributions across depths and seasons at station M1 **(A)** and M2 **(B)**. Only groups with at least 2% relative abundance in a sample are presented. Proteobacteria, Thaumarchaeota, and Euryarchaeota are split into various subclades for highlighting intra-phylum differences in abundance patterns. For Proteobacteria, Class-level identity is provided in brackets to each clade label in the legend. Blank columns in each panel represent missing samples. Also note that 500 m depth was not sampled in 2014. Winter months in 2014 were also not sampled due to logistical reasons.

**FIGURE 4:**
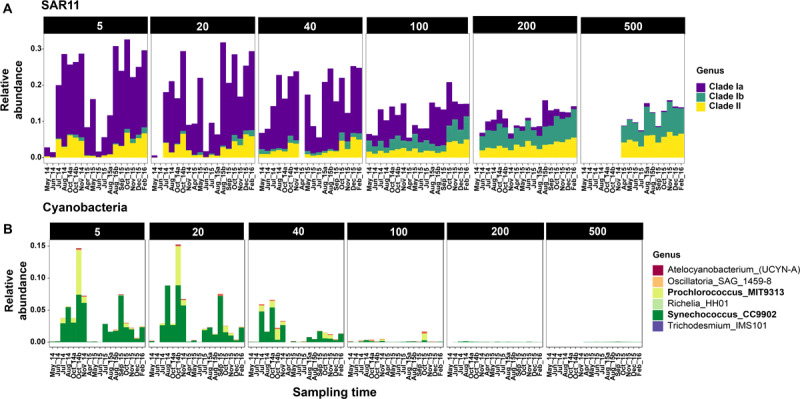
Relative abundances of SAR11 **(A)** and Cyanobacteria **(B)** clades across sampling time points at Station M1. Only selected depths are shown. Complete depth profiles for both stations are presented in Supplementary Figure S6 (SAR11) and Supplementary Figure S7 (Cyanobacteria). Note that the winter months in 2014 were not sampled due to logistical reasons.

Seasonal differences in abundances were more pronounced at finer taxonomic levels (Figure 3). At each depth group, distinct microbial groups appeared to correlate with seasonal hydrologic changes (Supplementary Table S2). Alphaproteobacteria (primarily SAR11 clades I and Rhodobacterales) was the most abundant class overall (87% of the total dataset); and showed clear within-clade temporal variability (Figure 3). SAR11 clades I and II were most abundant during the oceanic and winter seasons, and together accounted for <10% of the community in surface waters during the upwelling period (Figures 3, 4A and Supplementary Figure S6). As observed in SAR11 ecotype distributions across marine environments (e.g., Vergin et al., 2013), clade I was more abundant than clade II in the upper 100–200 m; however, at 500 m, both clades had similar relative abundances with little temporal variation (Figure 3 and Supplementary Figure S6). Within clade I, subclades Ia and Ib had contrasting abundances along the depth gradient: while Ia was primarily observed in depths above 80 m and exhibited seasonal changes in relative abundance, clade Ib became more dominant below 80 m, and was less influenced by temporal changes (Figure 4A and Supplementary Figure S6). Rhodospirillales and Rhodobacterales, two other major alphaproteobacterial groups, were also dominant in shallow waters (Figure 3) – Rhodospirillales on average accounted for 0.5% of the total community, whereas Rhodobacterales constituted up to 4.6% of the community. Both groups had contrasting abundance patterns through time: Rhodospirillales co-occurred with SAR11 clades post upwelling, whereas Rhodobacterales were most abundant during the upwelling season (Figure 3).

While Alphaproteobacteria dominated the proteobacterial fraction in shallow depths, Gamma- and Deltaproteobacteria became increasingly more abundant with depth. At 40 m and above, SAR86, Oceanospirillales, and Cellvibrionales were the dominant gammaproteobacterial groups, even though their combined abundances were 3–5 times smaller than co-occurring alphaproteobacterial groups (Figure 3). Below 40 m, Thiomicrospirales (including the SUP05 cluster) became the dominant gammaproteobacterial genus, with peak relative abundances of up to 34% of the community at 100 and 200 m depths. The only deltaproteobacterial genus that comprised at least 2% of the community across samples was the SAR324 clade (Marine group B). Although SAR324 were rarely detected in shallow depths, they were prominent members of the community at and below 80 m, with abundance maxima at 500 m (24% of the community in February 2016; Figure 3).

Cyanobacteria were abundant members of the community only during the oceanic and winter seasons. For example, in October 2014, Cyanobacteria accounted for 27% of the community at 5 m at M1 (Figure 3). Over 98% of the cyanobacterial SVs were classified as Synechococcales at the Order level (Figure 4B and Supplementary Figure S7); and predominantly constituted by *Synechococcus* CC9902 and *Prochlorococcus marinus* (strain MIT 9313) at the genus level. While *Synechococcus* were present throughout the year except during upwelling in surface waters, the *Prochlorococcus* SVs were observed only during late summer and fall (Figure 4B). Abundance peaks for SAR11 and picocyanobacteria in shallow depths coincided within the same temporal window (selected depths from M1 are presented in Figure 4; complete temporal profiles for each depth are presented in Supplementary Figures S6, S7), highlighting their adaptation for the relatively more oligotrophic conditions post-upwelling.

Most heterotrophic bacteria exhibited seasonally varying abundances, particularly within the top 40 m of the water column. Bacteroidetes (primarily Flavobacterales; Supplementary Figure S8) were among the most abundant taxa in shallow depths, often accounting for up to 50% of the SVs in the upper 40 m. Their abundance patterns mirrored that of SAR11 clade I and Cyanobacteria: highest relative abundances were observed during the upwelling season at both stations (Figure 3). Abundance peaks of Verrucomicrobia and Bacteroidetes coincided in shallow waters (Figure 3), and the two groups co-varied across all depths (strong abundance correlations with coefficients ranging from 0.94 to 0.99). An unclassified verrucomicrobial group (classified as ‘Arctic97B-4_marine_group’ at the Order level) appeared to be abundant at 500 m depths across seasons (particularly at M2; Figure 3). Finally, *Ca*. Marinimicrobia were more abundant deeper in the water column although a few SVs were dominant in shallow waters during the oceanic and winter seasons (Figure 3).

All archaeal SVs in the dataset were classified as either Euryarchaeota or Thaumarchaeota. Euryarchaeota had similar relative abundances across depths, comprising 0.5–1.5% of the community at each depth. At the Class level, euryarchaeal SVs were classified as either Thermoplasmata (Marine Group II and III) or Halobacteria (Supplementary Figure S9). Marine Group II accounted for 94% of the total euryarchaeal abundance, followed by MG III (5% of Euryarchaeota). Halobacteria comprised only up to 1% of the total euryarchaeal abundance, and were predominantly found below 40 m (Supplementary Figure S9).

Thaumarchaeota were a major fraction of the overall dataset, comprising 24.16% of the total community overall. At the genus level, a *Nitrosopumilus*-like group was the most abundant across all depths. An unclassified genus-level clade became particularly more abundant with depth, accounting for up to ∼90 % of thaumarchaeal abundance at 500 m (Figure 3). Top BLAST hits for SVs in this clade were sequences from the ‘deep’ water column B (WCB) group of Thaumarchaeota. The third *Nitrosopelagicus*-like (i.e., water column A) group was primarily found at 80 or 100 m, and accounted for 16.2 and 20% of the total thaumarchaeal abundance at M1 and M2, respectively. These results directly compare to the more in-depth, OTU-based analysis of thaumarchaeal 16S rRNA gene sequences in the MB time-series, as presented in Tolar et al. (2020).

### Taxonomic Composition and Functional Potential in Metagenomes and Metatranscriptomes

**FIGURE 5:**
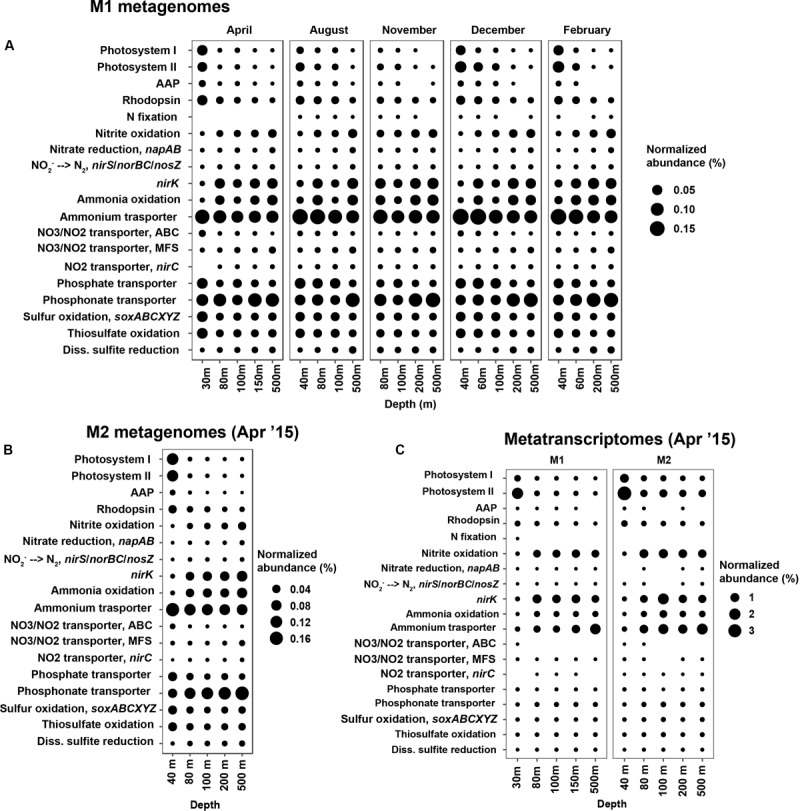
Normalized abundances of selected metabolic pathways/genes in the metagenomes and metatranscriptomes. Both color and size mapping correspond to the normalized abundance of each pathway or gene. **(A)** metagenomes from station M1, all collected in 2015; **(B)** metagenomes from station M2, representative of the upwelling season; and **(C)** metatranscriptomes from M1 and M2, collected during the upwelling season in April 2015. KO terms used for estimating relative abundances of each pathway are presented in Supplementary Table S1. Pathway/gene abbreviations: AAP, aerobic anoxygenic photosynthesis, *nirK*, nitrite redcutase.

Overall, taxonomic composition of the metagenomes and metatranscriptomes aligned with the 16S rRNA amplicon-based analysis (Supplementary Figure S10). In order to explore seasonal or depth-based changes in metabolic potential, we examined the abundance and expression of a selected set of key metabolic pathways across the metagenomes and metatranscriptomes (genes/pathways examined are presented in Supplementary Table S1). As would be expected, phototrophy-related genes had the highest abundance and expression in the shallow photic depths (30 and 40 m at both stations; Figure 5). At M1, for which we had a time-series of metagenomes, seasonal differences were apparent in the abundances of phototrophy genes (Figure 5A): both photosystem (PS)-I and PS-II were relatively less abundant at the onset of the oceanic season in August, as well as in November as the system transitions into the winter Davidson Current period. Potential for photoheterotrophy was present across all metagenomes and showed similar abundance trends as PS genes (Figure 5). Temporal changes in the abundances of aerobic anoxygenic phototrophy (AAP) genes aligned with the relative abundances of Rhodobacterales and Cellvibrionales, which are known to encode AAP genes (Imhoff et al., 2018).

Potential for nitrogen fixation was found in metagenomes representing non-upwelling periods at M1 (Figure 5A). There was no evidence for *nifH* gene transcription during the upwelling period (Figure 5C; although *nifD* and *nifK* transcripts were found in the M1 30 m metatranscriptome). Normalized abundances of all dissimilatory nitrogen cycle pathways appeared to increase with depth (Figure 5). Of particular importance is the nitrification pathway, which was most abundant below 40 m (Figures 5A,C). Abundance patterns of *nirK* across depths aligned with those of the core archaeal ammonia oxidation genes (i.e., ammonia monooxygenase subunits and hydroxylamine oxidoreductase from ammonia-oxidizing bacteria, all belonging to the genus *Nitrosomonas*, only accounted for 0.14% of the community overall). Nitrite oxidation genes had similar abundance and expression distributions as the genes for ammonia oxidation (Figure 5).

Among the transporter pathways considered, ammonium transporter (*amtB*) was one of the most abundant across all metagenomes (Figure 5). Abundance of *amtB* genes decreased with depth, whereas transcription appeared to be higher deeper in the water column (Figure 5C). The different types of nitrate/nitrite transporters compared here showed contrasting abundance patterns with depth: while ABC-type nitrite/nitrate transporter gene (encoding NrtABCD) abundances decreased with increasing depth, MFS family NarK transporter genes increased in abundance from 30 to 500 m. These trends were preserved across all timepoints at M1 (Figure 5A). Transcription of Nrt genes was limited compared to NarK during the upwelling season (Figure 5C). Similar depth trends were observed in the abundances of phosphate versus phosphonate transporter genes – phosphate transporters (*pstABCS*) were more abundant at 100 m and above, whereas phosphonate transporter (*phnCDE*) abundances (in both metagenomes and metatranscriptomes) increased steadily with depth (Figure 5).

## Discussion

In the present study, we identified clear seasonal abundance patterns and depth-differentiation within microbial assemblages in the MB upwelling system, in accordance with the dynamically changing hydrology of the bay. Our results specifically pointed to sequential changes in community composition across the three oceanographic seasons characteristic of the MB system.

### Depth-Differentiation Within Bacterioplankton Assemblages

Across all time points, strong vertical gradients existed in physicochemical variables (Supplementary Figures S1, S11–S13) and, as a result, distinct depth stratification was evident in the distribution of microbial populations, aligning with previously reported trends in pelagic communities (e.g., DeLong et al., 2006; Brown et al., 2009; Agogué et al., 2011; Walsh et al., 2016). Taxonomic richness and evenness were lowest at the shallow photic depths, consistent with prior studies that observed increasing alpha diversity with depth (e.g., Soupene et al., 2002; Ghiglione et al., 2012; Walsh et al., 2016). At both stations, richness and evenness peaked below the euphotic zone (80–100 m) and stayed relatively invariant down to 500 m (Figure 1). This potentially reflects gradients in nutrient availability and composition: high primary productivity at the surface potentially creates a diverse suite of organic matter to support different heterotroph growth strategies in intermediate waters. The seasonal variability at 500 m depth appeared to be explained primarily by phaeopigment levels (Figure 2F and Table 1), which potentially relates to increased particle flux from surface waters during the upwelling periods.

Phototrophic organisms, including Cyanobacteria and putative anoxygenic phototrophs such as Rhodobacterales and Cellvibrionales, decreased in abundance with depth (Figure 3 and Supplementary Figure S7). As would be expected, this aligned with the decreasing relative abundances of aerobic anoxygenic phototrophy-related genes (AAP) with depth (Figures 5A,B). AAP gene abundances correlated with the relative abundances of Rhodobacterales and Cellvibrionales with *R*^2^ values of 0.94 and 0.76, respectively (Supplementary Figure S14). The relatively steeper decrease in photosystem gene abundances with depth compared to rhodopsin genes (Figure 5A) likely relates to differential responses of photoheterotrophs and photosynthetic organisms to light availability.

Aerobic heterotrophs such as SAR11 and SAR86 were particularly abundant in shallow depths, along with Rhodobacterales, Cellvibrionales (Gammaproteobacteria) and Flavobacteria, which are known bloom-associated groups (Buchan et al., 2014). More metabolically versatile groups such as Thiomicrospirales (SUP05, ARCTIC96BD-19 clades of sulfur oxidizers with high metabolic plasticity; ref. Murillo et al., 2014) and *Ca*. Marinimicrobia (implicated in sulfur and nitrogen transformations; ref. Hawley et al., 2017) became more abundant fractions of the community below the photic zone. Indicating further turnover in community composition with depth, Thaumarchaeota and Nitrospinae also increased in abundance below 40 m, together constituting up to ∼35% of the community at 500 m (Figure 3). These abundance profiles likely indicate a combined effect of different depth-varying physicochemical components including light, organic matter composition (labile vs. more recalcitrant carbon), oxygen levels, as well as various co-occurrence/interaction relationships among microbial clades.

Differential depth-distribution of certain microbial groups has previously been reported in MB waters, albeit with limited spatiotemporal and phylogenetic resolution. For example, in a large-insert BAC library analysis targeting typical marine Gamma- and Alphaproteobacteria across four depths in MB, Suzuki et al. (2004) reported differential depth distribution within SAR11, Rhodobacterales, and gammaproteobacterial clades. More recently, Rich et al. (2011) reported clear community separation between photic and subphotic depths in MB (i.e., surface and 30 m depths vs. 200 m), based on a time-series analysis of genome proxy microarrays targeting 268 genotypes across typical marine bacterioplankton. Their time-series spanned 4 years at station M1, but despite the strong upwelling response observed for certain abundant target taxa (Roseobacter and SAR86), they did not find a clear correlation between the oceanographic seasons and microbial community structure. In contrast, our high-resolution profiling of the MB bacterioplankton community substantially expands the general findings of the above two studies by showing not only depth-based segregation across clades, but also seasonally recurrent abundance patterns.

### Microbial Community Dynamics Across Hydrographic Seasons

We found significant changes in community composition and metabolic potential associated with seasonal transitions in MB, which have been largely unrecognized prior to this study. Seasonal patterns were particularly dominant in the upper 40 m of the water column (Figures 1, 2). Lowest alpha diversity was observed during the spring/summer upwelling season, while oceanic and winter seasons harbored more diverse bacterioplankton assemblages, particularly in shallow depths. Many of these abundance patterns likely result from heterotrophic responses to increased primary production during upwelling. Bacteroidetes, Rhodobacterales, and Gammaproteobacteria (predominantly Cellvibrionales) that show abundance maxima during the spring/summer upwelling season are known degraders of phytoplankton-derived polysaccharides and osmolytes (Buchan et al., 2014). Similar abundance maxima of heterotrophic bacteria have been observed in other coastal systems during bloom conditions (e.g., Alderkamp et al., 2006; Buchan et al., 2014; Lindh et al., 2014; Teeling et al., 2016). Even during high-nutrient conditions, at least some of these heterotrophic groups supplement their energy requirements by using a light-driven proton pump, as we saw the transcription of AAP genes in the metatranscriptomes collected during the upwelling season (Figure 5C).

Fall phytoplankton blooms occasionally observed during the oceanic season in MB are suggested to be compositionally distinct from those in spring/summer – while diatoms dominate the upwelling blooms, fall blooms are largely composed of dinoflagellates (Garrison, 1979). Rich et al. (2011) observed no change in post-bloom bacterial communities with respect to the dominant phytoplankton in each bloom season. Our data, however, points to potential differences in bloom-associated groups as indicated by the clear dominance of Rhodospirillales and SAR11 during fall, and Flavobacteria and Rhodobacterales during spring/summer (Figure 3). Ascertaining whether these occurrence patterns are truly tied to compositional changes in bloom communities would require a better mechanistic understanding of phytoplankton-heterotroph interactions, alongside continued temporal monitoring of community composition.

Alpha-diversity comparisons (Figure 1) suggested potential temporal differences between the two stations. While upwelling-induced changes in shallow-depth community composition at M1 persisted into the oceanic season, the more offshore station M2 experienced more distinct transitions between the three seasons. Peaks in alpha-diversity indices (across all depths) in the oceanic and winter season (Figure 1) may be explained by heterotrophic responses to bloom decline. As the bay transitions into the oceanic season, phytoplankton growth draws down inorganic nutrient concentrations to a minimum (as seen in Supplementary Figures S4, S11–S13). The low-nutrient conditions likely favor oligotrophic groups such as SAR11 and picocyanobacteria, which appear to dominate shallow waters during the oceanic season (Figure 3 and Supplementary Figures S6, S7). Studies in other marine systems have also reported peaks in diversity during the winter season, usually attributed to increased water column mixing (e.g., Gilbert et al., 2009; Chow et al., 2013; Garcia et al., 2015; Hernando-Morales et al., 2018). In Monterey Bay, this pattern may result from the transport of offshore taxa under post-upwelling conditions. As discussed previously, the upwelling-to-oceanic transition period is characterized by the California Current moving into the bay to replace the sinking upwelled water. It is likely that the inflowing offshore waters bring in open-ocean bacterioplankton such as SAR11 and *Prochlorococcus*, which then thrive in the relatively nutrient-poor conditions during bloom decline. Indeed, Skogsberg (1936) and Bolin and Abbott (1960) defined the oceanic season for MB based on both surface water characteristics and the presence of offshore plankton groups, although their analyses did not include direct characterization of the archaeo- and bacterio-plankton components.

### Transporter Abundances Change Across Depths and Seasons

Abundances of genes involved in key metabolic pathways largely aligned with changes in microbial community composition. Particularly intriguing was the spatiotemporal variation in transporter abundances, which likely reflected the dynamic nutrient profiles and the associated changes in community structure across spatiotemporal gradients. Considering phosphorus (P) transport pathways, the 500 m depth always had 1–2 fold more phosphate compared to the shallowest depth (Supplementary Figure S12) and, accordingly, the abundance of phosphate transporters (*pstABCS*) decreased vertically downward. In contrast, transporters for phosphonate (*phnCDE*), an alternative P source for bacterioplankton (reviewed in Villarreal-Chiu, 2012), became more abundant with depth. Transcription of phosphonate transporters was observed during the upwelling period, even though this time point had the highest phosphate concentrations across all depths (Supplementary Figure S12). Assuming that phosphonate acts as an alternative P source, these results confirm that phosphonate catabolism may be independent of inorganic phosphate concentrations, as suggested previously (Villarreal-Chiu, 2012). Furthermore, the imported phosphonate may not necessarily be utilized as a P source; it has been suggested that phosphonates may be processed via an anabolic route and incorporated into various macromolecules (Villarreal-Chiu, 2012). The abundance patterns observed here are intriguing either way; it remains to be determined if these patterns are linked to a phylogenetic signal as opposed to *in situ* P levels (e.g., increasing abundances of Thaumarchaeota with depth, since some thaumarchaeal clades are known to encode phosphonate transporters; Swan et al., 2014).

Ammonium transporter (*amtB*) genes were particularly abundant across all metagenomes; highest normalized abundances were observed in shallow depths (Figures 5A,B). Interestingly, transcription of *amtB* appeared to progressively increase with depth (Figure 5C), potentially reflecting the vertically decreasing ammonia/ammonium concentrations as seen in Supplementary Figure S11. As in the case of phosphonate transporters, another explanation for the higher expression of *amtB* at depth might be the increasing relative abundance of Thaumarchaeota with depth. Similar expression profiles of ammonia-oxidation related genes (*nirK*, *amoABC*, and *hao*) and *amtB* (Figure 5C) further corroborate this possibility.

Differential depth-distribution of ABC-type vs. MFS nitrate/nitrite transporter genes potentially indicates differences in community composition with depth. Dissimilatory nitrate-reducing bacteria primarily possess NarK, belonging to the MFS superfamily of transport proteins (Alvarez et al., 2018); whereas nitrate-assimilating organisms typically possess ATP-requiring ABC-type transporters (Moir and Wood, 2001). The distribution of *narK* with depth concurs well with the abundance patterns of other denitrification genes (Figure 5). We do not expect active denitrification in the depths sampled here, given the water column was well-oxygenated during the study period (lowest observed oxygen levels were observed at 500 m; which never fell below 0.35 ml/L; Supplementary Figure S13). However, localized pockets of anoxia (e.g., phytoplankton aggregates or other particulate matter, particularly during the upwelling period), or a temporal separation between active oxygen generation and nitrate reduction could support denitrification in these waters.

In conclusion, we find clear spatiotemporal patterns in planktonic bacterial and archaeal community structure, reflecting the dynamic hydrography of the MB upwelling system. Temporal changes in community structure aligned with seasonal transitions, and variability was particularly pronounced in shallow depths (top 40 m of the water column). However, questions remain regarding how robust these patterns are across a longer time-series, especially since our sampling period coincided with the 2014–2016 northeast Pacific warm anomaly (Bond et al., 2015; Di Lorenzo and Mantua, 2016). Seasonal anomalies in nutrient profiles associated with upwelling and relaxation events in MB were significantly intensified during this period, leading to severe ecological perturbations such as the development of the largest ever-recorded toxic algal bloom (Ryan et al., 2017). We did not observe significant variability in microbial community structure between 2014 and 2015; however, the lack of baseline data for ‘normal’ ocean conditions precludes a meaningful comparison.

Recent modeling studies have suggested significant changes to the California Current System in response to a changing climate (Snyder et al., 2003; Xiu et al., 2018). Stronger alongshore winds are projected to facilitate increased upwelling intensity, leading to higher nutrient fluxes to shallow euphotic depths. This in turn is projected to cause a significant decline in oxygen levels, potentially leading to more frequent occurrences of hypoxia (Dussin et al., 2019). Importantly, however, we do not yet understand how the microbial community would respond to these changes due to lack of spatiotemporally resolved baseline data. This emphasizes the need for continued high-resolution monitoring of the bay ecology, not only to confirm the patterns we observed here, but also to predict the behavior of the system under projected changes in physicochemical conditions.

## Data Availability Statement

The datasets generated for this study can be found in the NCBI Sequence Read Archive (SRA) database under BioProject PRJNA488312 (SRA accession: SRP159037), Metagenome and metatranscriptome assemblies are available in IMG under the GOLD study ID Gs0099541.

## Author Contributions

LR processed and analyzed the data, and drafted the manuscript. BT and LR collected and processed samples, and acquired metagenome, metatranscriptome, and amplicon sequence datasets. FC facilitated sample acquisition and provided logistical support in the field. CF and FC designed the study and acquired funding. CF supervised the overall planning and execution of the project.

## Conflict of Interest

The authors declare that the research was conducted in the absence of any commercial or financial relationships that could be construed as a potential conflict of interest.
