# Supplementary material for: Depth-Differentiation and Seasonality of Planktonic Microbial Assemblages in the Monterey Bay Upwelling System
Source: Front Microbiol. 2020 May 25;11:1075. doi: 10.3389/fmicb.2020.01075 (PMC7261934; doi:10.3389/fmicb.2020.01075)
Supplement: Supplementary file 1 [file Data_Sheet_1.PDF]

Supplementary Material

Table S1: KO/COG terms used for examining the abundances of selected pathways across metagenomes and metatranscriptomes. Results are presented in Fig. 4.

**Pathway**	**Enzyme(s) and/or KOs**
Photosystem I	K02689, K02690, K02691, K02692, K02693, K02694, K02695, K02696, K02697, K02698, K02699, K02700, K02701, K02702
Photosystem II	K02703, K02704, K02705, K02706, K02707, K02708
Anoxygenic photosynthesis	PufLM; K08928, K08928
Rhodopsin	COG5524; K04641
Nitrogen fixation	NifHDK; K02588, K02586, K02591
Nitrite oxidation/nitrate reduction	NxrAB/NarGH; K00370, K00371
Nitrate reduction	NarL, NapAB; K00374, K02567, K02568
Denitrification (nitrate to dinitrogen)	NirS, NorBC, NosZ; K15864, K04561, K02305, K00376
Nitrite reductase (NirK)	K00368
Ammonia oxidation	AmoABC, Hao; K10944, K10945, K10946, K10535
Ammonium transporter	AmtB; K03320
NO3/NO2 ABC transporter	NrtABCD; K15576, K15577, K15578, K15579
NO3/NO2 MFS transporter	NarK; K02575
NO2 transporter	NirC; K02598
Phosphate transporter	PstABCS; K02036, K02037, K02038, K02040
Phosphonate transporter	PhnCDE; K02041, K02042, K02044
Sulfur oxidation	SoxABCXYZ; K17222, K17223, K17224, K17225, K17226, K17227
Dissimilatory sulfite reduction	DsrAB: K11180, K11180

Table S2: Taxa correlating with the Bakun upwelling index at each depth group across the two stations. The upwelling index was used as a proxy for seasonality in MB. Only $R^2 > 0.3$ are reported.

Depth group	Station M1		Station M2	
	Taxonomic group	R^2	Taxonomic group	R^2
5 - 20 m	Actinobacteria	0.561	SAR11 Clade_I (α)	-0.587
	SAR11 Clade_I (α)	-0.352	SAR 11 Clade_II (α)	-0.432
	SAR 11 Clade_II (α)	-0.415	SAR 11 Clade_IV (α)	0.535
	OCS116 (α)	0.556	Rhodobacterales (α)	0.334
	Rhodobacterales (α)	0.303	SAR116 (α)	0.502
	SAR116 (α)	-0.366	Nitrosopumilus (Thaumarchaeota)	-0.547
	Cellvibrionales (ɣ)	-0.542	Oceanospirillales (ɣ)	0.713
	Oceanospirillales (ɣ)	0.557	SAR86 (ɣ)	-0.467
	SAR86 (ɣ)	-0.458	Thiomicrospirales (ɣ)	-0.327
	Marinimicrobia	-0.541	Euryarchaeota	0.484
	Verrucomicrobia	0.361		
30 - 40 m	SAR 11 Clade_II (α)	-0.623	SAR11 Clade_I (α)	-0.605
	OCS116 (α)	-0.318	SAR 11 Clade_II (α)	-0.315
	Rhodobacterales (α)	0.321	OCS116 (α)	-0.828
	Nitrosopelagicus (Thaumarchaeota)	-0.312	Rhodobacterales (α)	0.4
	Oceanospirillales (ɣ)	0.685	SAR86 (ɣ)	-0.427
	SAR86 (ɣ)	0.321	Thiomicrospirales (ɣ)	0.333
	Thiomicrospirales (ɣ)	0.35	Synechococcus	-0.33
	Verrucomicrobia	0.328		
80 - 100 m	AEGEAN-169 (α)	0.858	Actinobacteria	0.922
	SAR324 (δ)	-0.47	AEGEAN-169 (α)	0.809
	Oceanospirillales (ɣ)	0.463	SAR11 Clade_I (α)	-0.415
	Thiomicrospirales (ɣ)	0.593	SAR 11 Clade_II (α)	-0.624
	Euryarchaeota	-0.392	Nitrosopelagicus (Thaumarchaeota)	-0.564
	Marinimicrobia	-0.402	Oceanospirillales (ɣ)	1
	Nitrospina	-1	Thiomicrospirales (ɣ)	0.706
			Marinimicrobia	-0.3
200 m	Actinobacteria	0.894	Actinobacteria	-0.334
	SAR 11 Clade_II (α)	-0.479	SAR 11 Clade_II (α)	-0.474
	Unclassified alphaprot.	-0.469	Nitrosopelagicus (Thaumarchaeota)	-0.464
	Nitrosopelagicus (Thaumarchaeota)	-0.416	SAR324 (δ)	-0.441
	Oceanospirillales (ɣ)	0.958	Euryarchaeota	-0.355
	Unclassified gammaprot.	0.506	Marinimicrobia	-0.445
	Nitrospina	-0.315	Nitrospina	-0.434
500 m	SAR11 Clade_I (α)	-0.534	SAR11 Clade_I (α)	-0.476
	SAR 11 Clade_II (α)	-0.556	SAR 11 Clade_II (α)	-0.564
	Unclassified alphaprot.	-0.527	Unclassified alphaprot.	-0.831
	SAR324 (δ)	-0.36	Bacteroidetes	-0.436
	Oceanospirillales (ɣ)	-0.89	Nitrosopumilus (Thaumarchaeota)	-0.443
	Thiomicrospirales (ɣ)	0.327	SAR324 (δ)	-0.705
	Marinimicrobia	0.381	Thiomicrospirales (ɣ)	0.532
	Nitrospina	-0.661	Euryarchaeota	0.504
	Thaumarchaeota genus	-0.41	Thaumarchaeota genus	-0.336
	Verrucomicrobia	1	Verrucomicrobia	-0.462

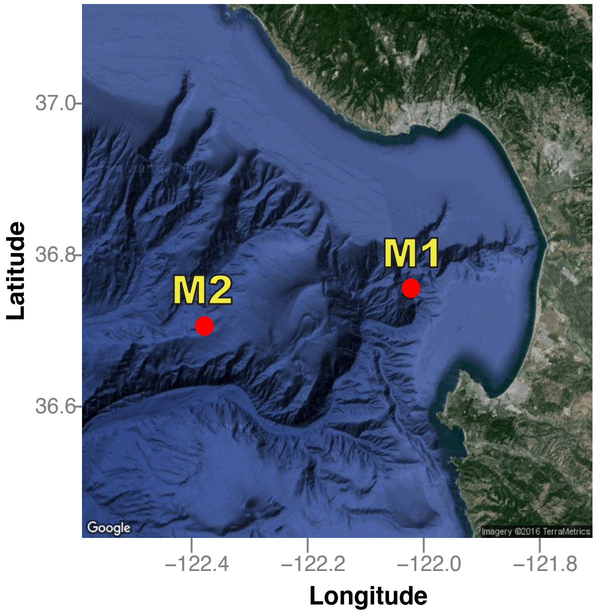

Figure S1: Map of the sampling stations in Monterey Bay. Station M1 is located ~20 km from shore while M2 is 72 km from shore.

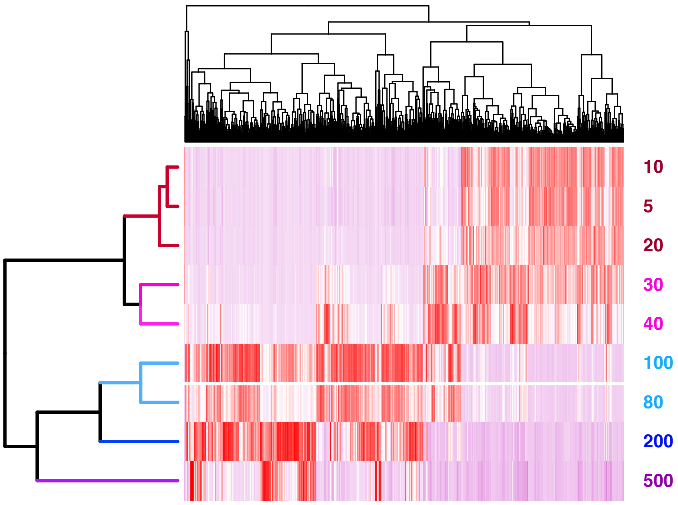

Figure S2: Results of hierarchical clustering performed on the Bray-Curtis dissimilarity (based on SV counts) between depths. Each column in the heatmap shows the relative abundance pattern of a unique SV while the rows represents sampling depths. Row color coding corresponds to the selected clusters (i.e., depth groups).

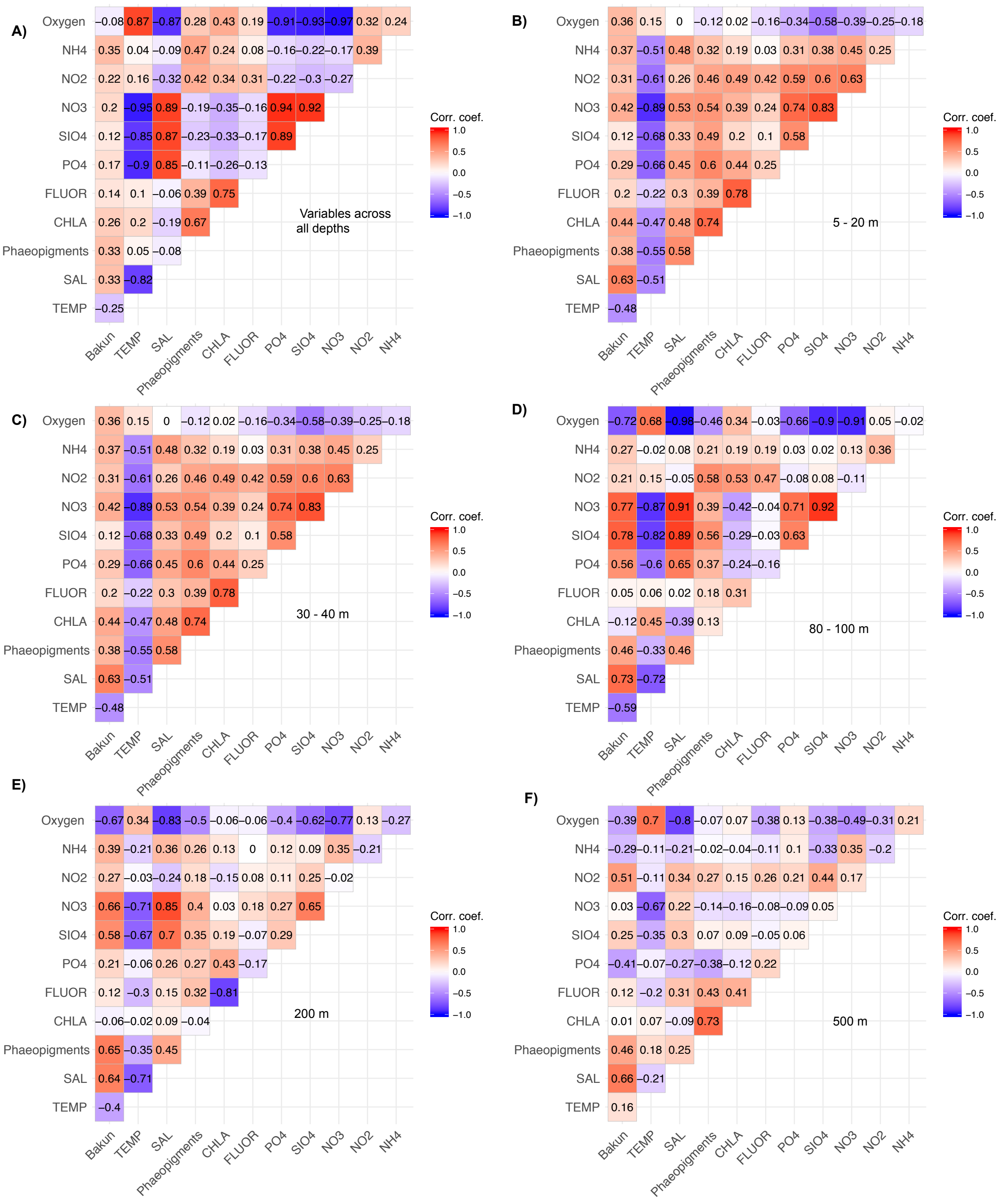

Figure S3: Correlation between measured environmental varaibles A) across the whole dataset, and B-F) within individual depth groups used in statistical analyses. At each depth category, redundant variables were dropped from statistical analyses if the correlation coeffient was > 0.9.

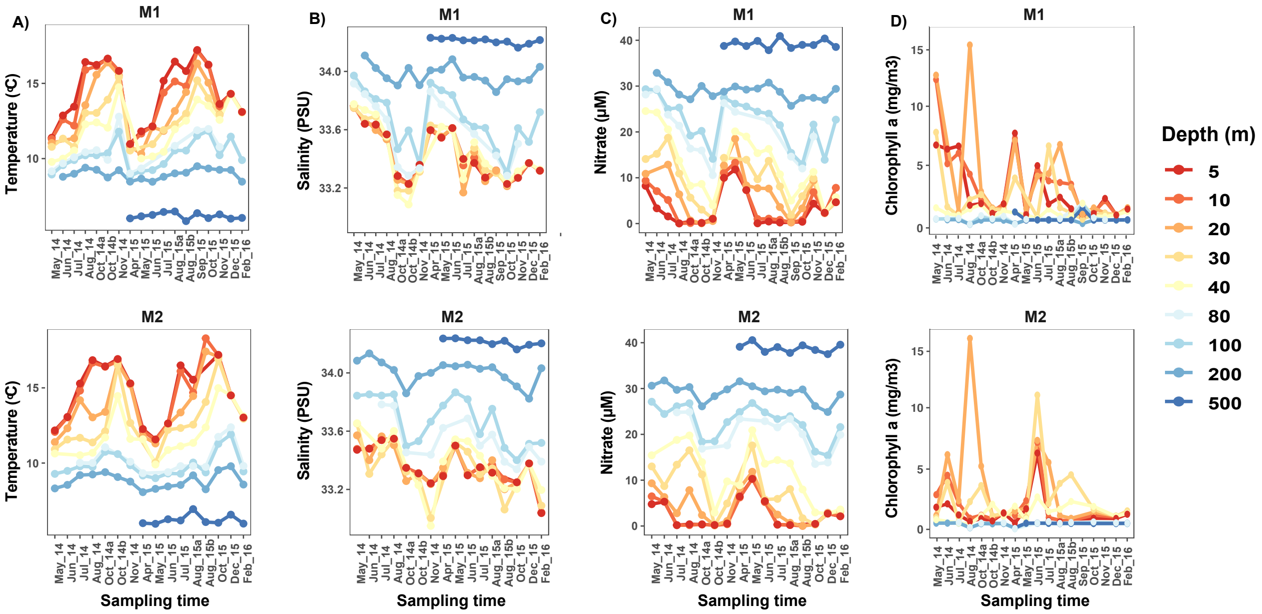

Figure S4: Temporal variation in A) temperature, B) salinity, C) nitrate and D) Chlorophyll a concentrations at the two stations.

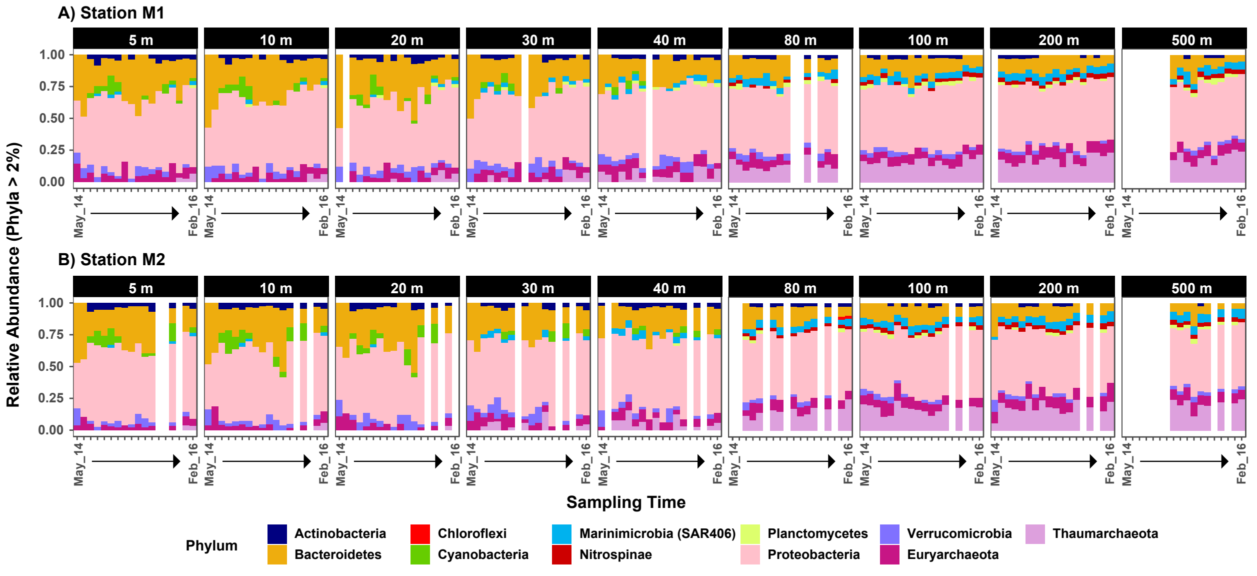

Figure S5: Spatio-temporal variation in phyla relative abundance at station M1 (A) and M2 (B). For simplification, sequence variants with < 2% abundance across all samples were filtered out.

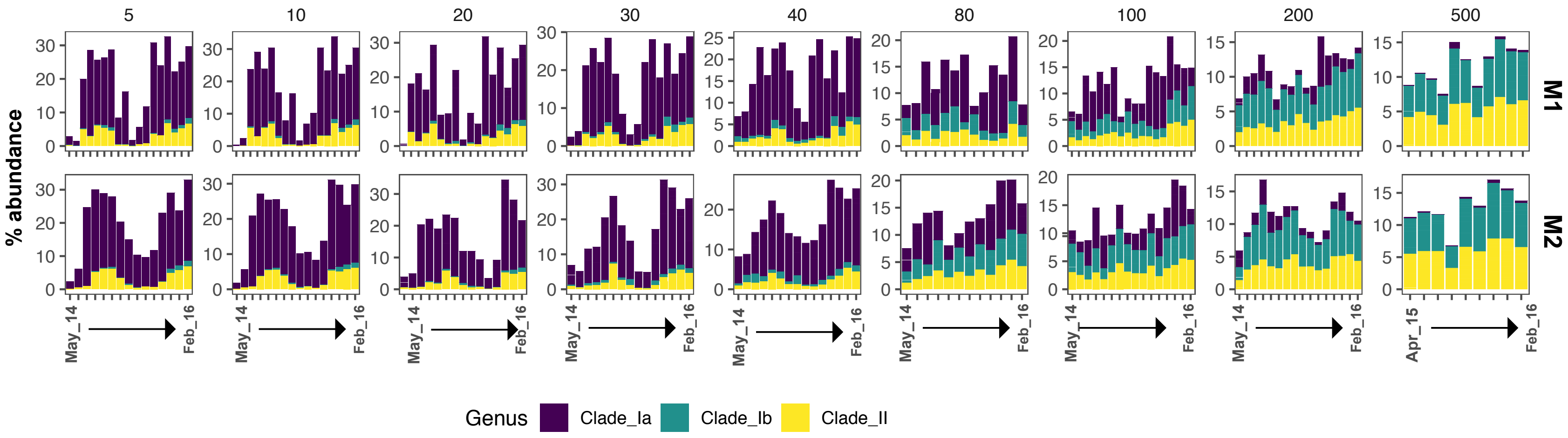

Figure S6: SAR11 clades across depths at stations M1 and M2. Clade I has been split into the two subclades Ia and Ib. Abundance is presented as a percentage of the total community.

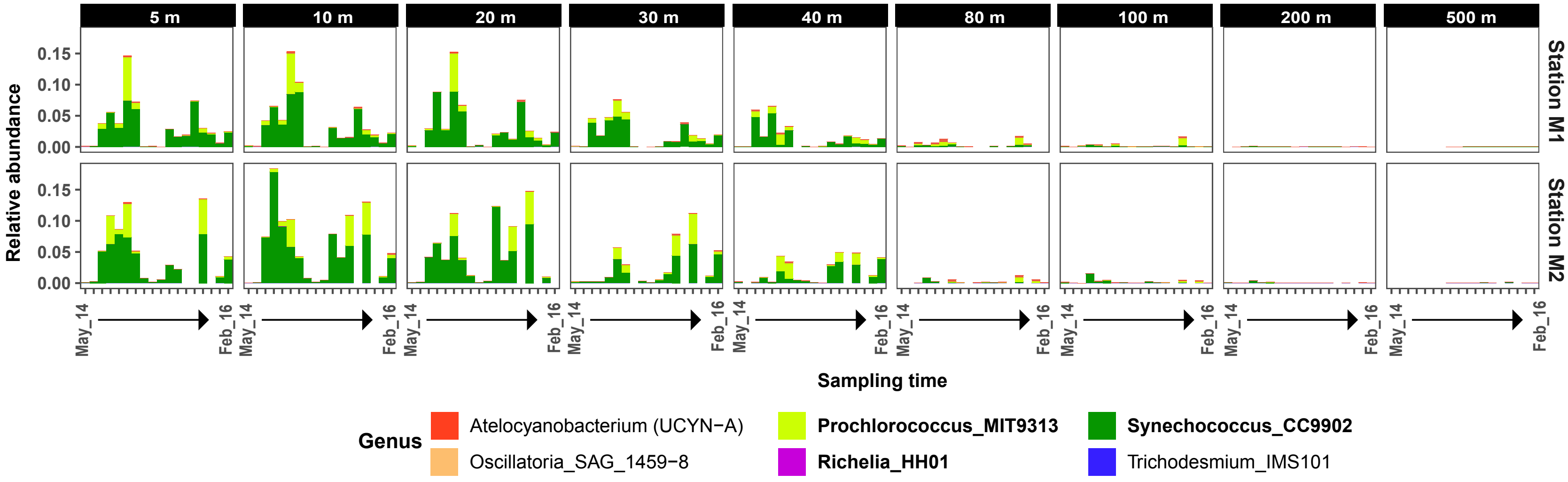

Figure S7: Cyanobacterial groups across depths at the two stations. Genus-level classifications are shown.

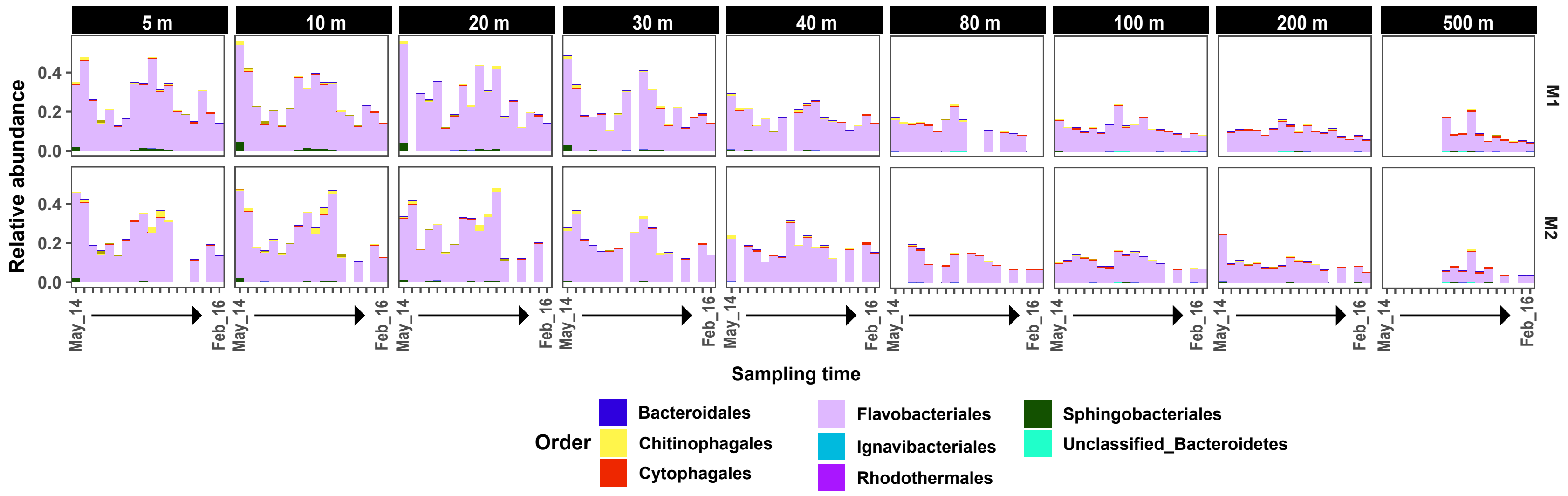

Figure S8: Abundances of Bacteroidetes Orders across depths at the two stations.

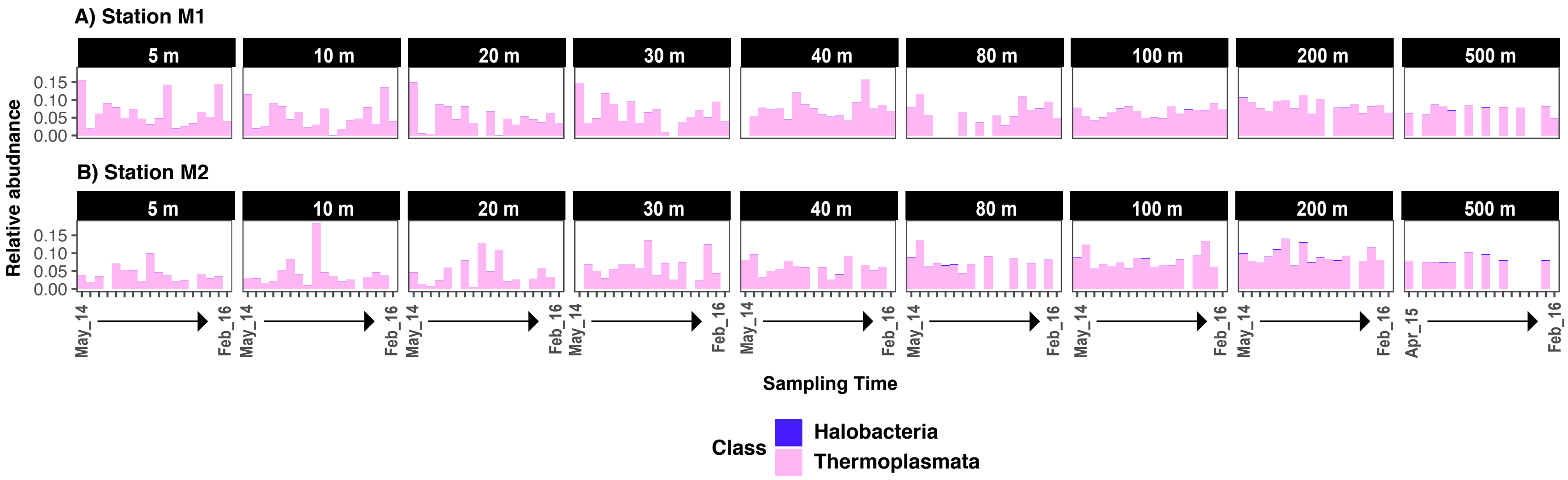

Figure S9: Within-clade variabliity in Euryarchaeota clades across depths at A) station M1 and B) station M2.

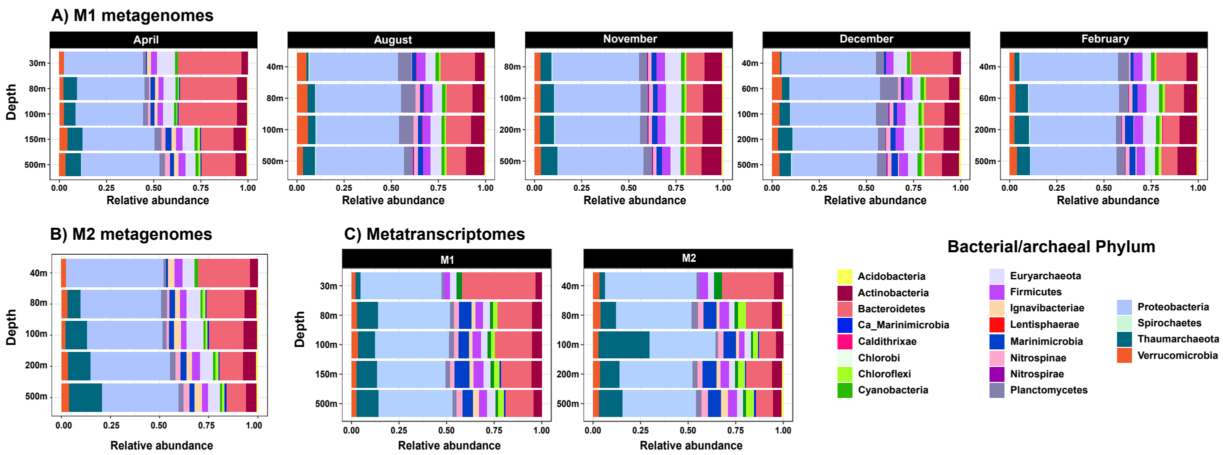

Figure S10: Taxonomic composition of short reads in the metagenomes and metatranscriptomes. Panel A presents relative abundance of major archaeal/bacterial phyla with depth, across seasons at station M1. Panel B shows the phyla abundance profiles at station M2 across depths (all collected during the upwelling period in April 2015). In panel C, taxonomic composition and dynamics inferred based on metatranscriptome short reads is presented. All 10 of the metatranscriptomes were collected in April 2015, during the upwelling period.

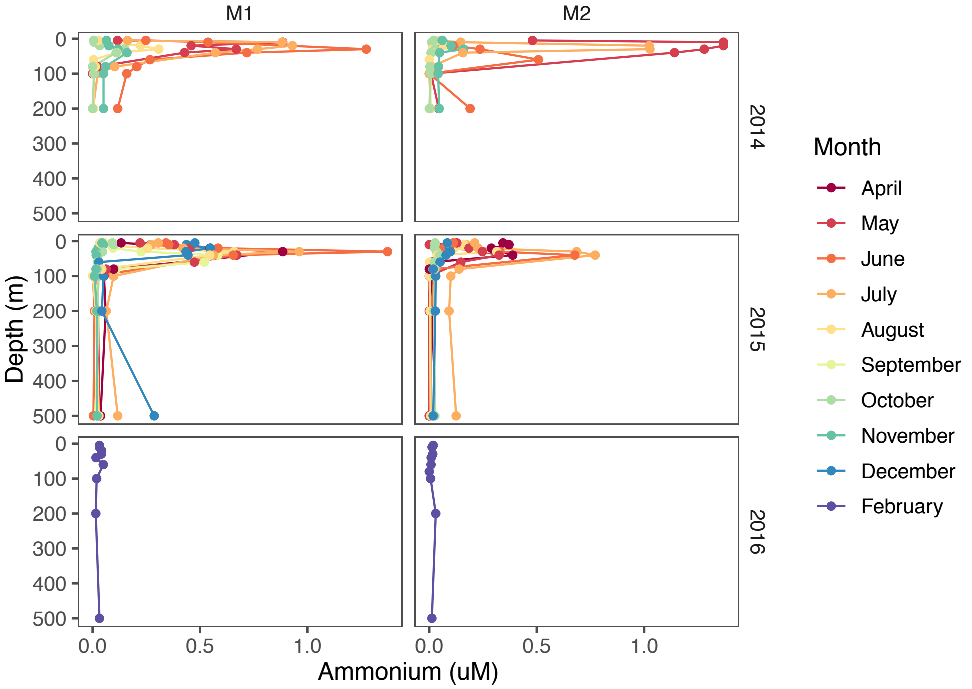

Figure S11: Temporal and depth-variation of ammonium concentrations (μM) at the two stations.

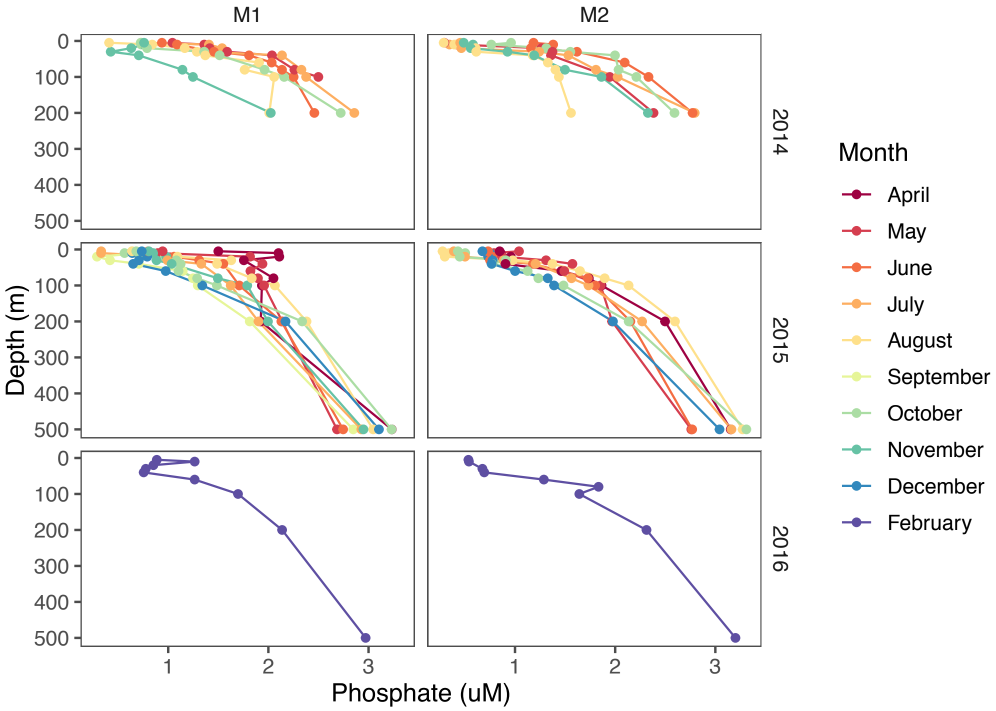

Figure S12: Temporal and depth-variation of phosphate (μM) concentrations at the two stations.

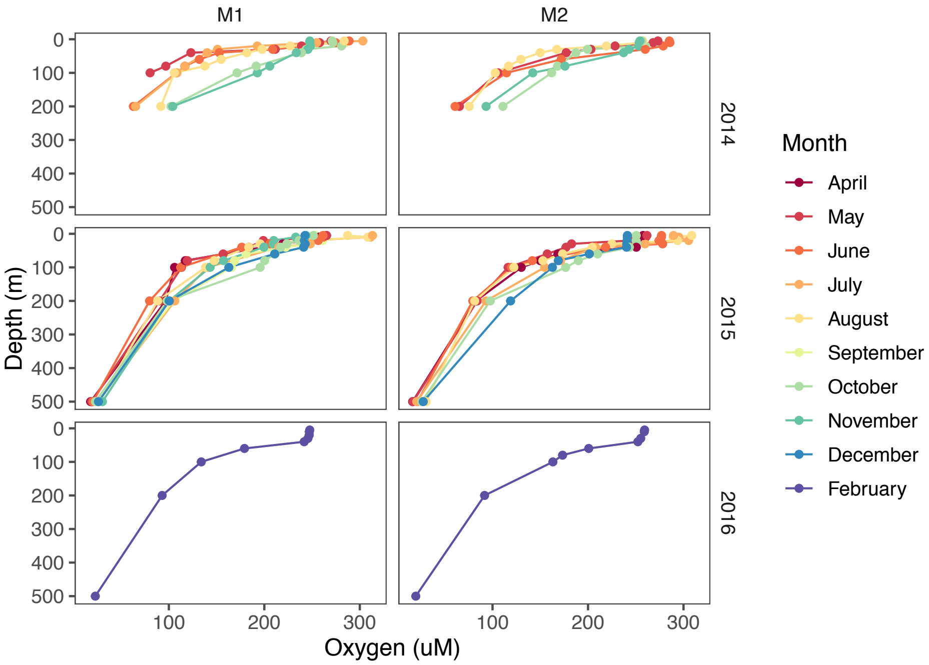

Figure S13: Temporal variability in oxygen concentrations (ml/L) across depths at the two stations

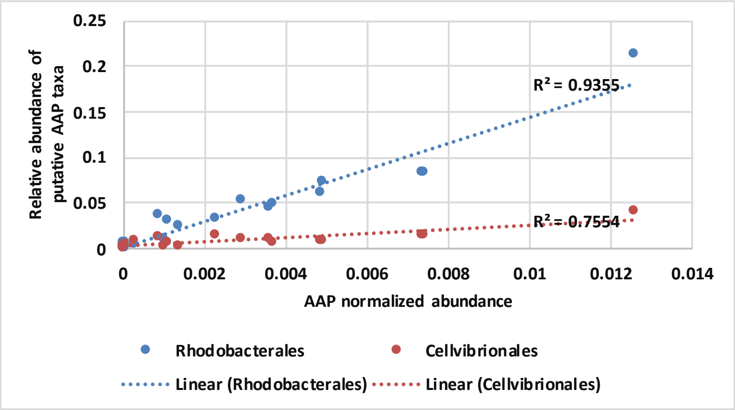

Figure S14: Regression plot showing the correlation between the relative abundances of putative AAP taxa in the 16S rRNA amplicon dataset and the normalized abundances of AAP genes in the metagenomes.

frontiers
